# Identification of Candidate epitopes from nation-enriched sequences in the African swine fever virus genomes

**DOI:** 10.1371/journal.pone.0354143

**Published:** 2026-07-23

**Authors:** Sieun Kim, Eun Bae Kim

**Affiliations:** 1 Department of Applied Animal Science, College of Animal Life Sciences, Kangwon National University, Chuncheon, Republic of Korea; 2 Institute of Animal Life Science, Kangwon National University, Chuncheon, Republic of Korea; Chung-Ang University, KOREA, REPUBLIC OF

## Abstract

African swine fever virus (ASFV) is a highly lethal pathogen that causes African swine fever (ASF) and is found in Africa, Europe, and Asia. In addition, extensive regional genetic variation poses significant challenges for vaccine development. In this study, we aimed to identify amino acid sequence fragments that are commonly enriched within ASFV strains from each country and evaluate their potential as epitopes. Amino acid sequences of genomes were segmented into overlapping 9-, 12-, 15-, and 20-mer peptides; selected fragments enriched in countries with at least ten available genomes; and cytotoxic T lymphocyte (CTL), helper T lymphocyte (HTL), and linear B lymphocyte (LBL) epitope predictions, followed by antigenicity assessment. Genomes from Italy yielded the highest number of fragments across all peptide lengths, whereas those from Russia showed a unique pattern in which fragment counts decreased as peptide length increased. From the five countries analyzed, 136 CTL, 95 HTL, and 97 LBL candidate epitopes were identified, of which 39 CTL, 18 HTL, and 75 LBL epitopes were predicted to be antigenic. The identified epitopes originated from structural (*n* = 24), replication/transcription (*n* = 11), and multigene family (*n* = 38) proteins. The resulting epitope library provides a comprehensive resource for the selection of multi target vaccines and diagnostic candidates. These findings provide a foundation for the design of region-specific vaccines and can be broadly applied for the future development of ASFV vaccines and diagnostic tools.

## Introduction

African swine fever (ASF) has recently gained worldwide attention as a serious threat to the livestock and wildlife industries [1]. The disease affects wild boars and domestic pigs, causes high fever, vomiting, and other clinical signs, and is characterized by a very high mortality rate [2]. These pathological outcomes are caused by African swine fever virus (ASFV), a double-stranded DNA virus with a genome size of approximately 170–194 kb [3].

Based on genetic analysis, 24 ASFV genotypes have been identified, with genotypes I and II responsible for most of the documented spread [4]. Genotype I was once widespread across Europe. However, apart from Italy, it has now been eradicated in nearly all European countries [5]. Genotype II, initially confined to Africa, rapidly spread to Europe and Russia after being first detected in Georgia in 2007 [6]. In 2018, genotype II entered China and began spreading throughout Asia; ongoing outbreaks have been reported across Europe, Asia, and Africa [7].

Since the 1960s, numerous studies have attempted to develop vaccines against ASFV. However, a commercially available vaccine has yet to be developed. This situation contrasts sharply with other DNA‐virus infections, such as smallpox, hepatitis B, and human papillomavirus, for which effective vaccines have successfully prevented disease [8]. The major obstacle is the structural complexity of ASFV, which encodes an extensive repertoire of proteins, many of which are dedicated to immune evasion, thereby undermining conventional vaccine approaches [9].

Furthermore, genomic-level variations were observed following the regional introduction of ASFV [10]. When compared genotype II ASFV strains isolated in Europe and Asia, single nucleotide variations (SNVs) had occurred in genes involved in immune suppression and evasion. As a result, the European and Asian strains formed distinct clusters. With the continual emergence of new strains and progressive accumulation of genetic divergence, it is unlikely that a single vaccine will confer equivalent protective efficacy against all variants.

Accordingly, vaccine design should be informed by strategies that account for regional variations and genetic differences. In this study, we analyzed all ASFV strains reported to date, extracted amino acid sequence fragments that are enriched within each country, and assessed whether any of these fragments harbored sequences with potential utility as immunogenic epitopes.

## Materials & methods

### Collection of the ASFV genome and country information

**Fig 1:**
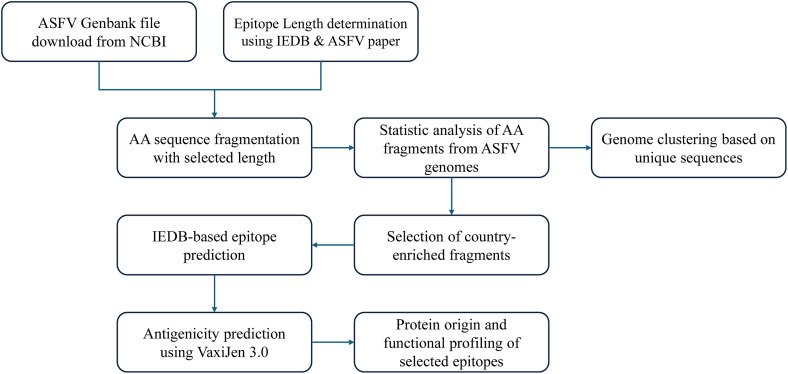
Flowchart of the evaluation of ASFV nation-enriched fragments as epitope. The overall analytical procedure is summarized in the flow chart.

**Fig 2:**
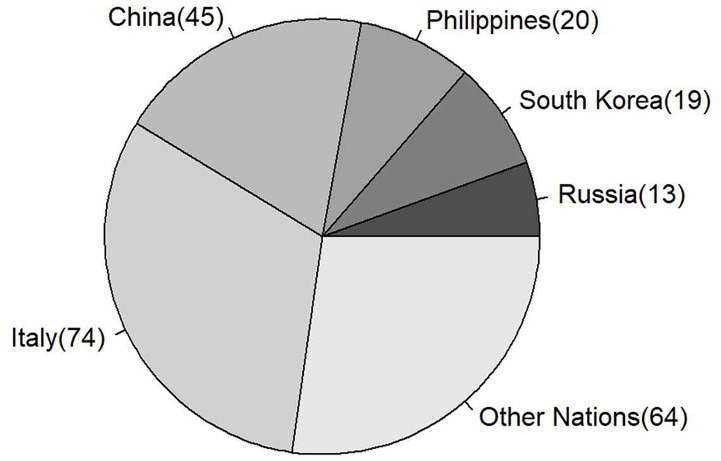
Country distribution of strains. The number of strains detected per country was compiled from 235 genome sequences. Countries with ten or fewer detected strains (Benin, Burundi, Estonia, Georgia, Hungary, Indonesia, Malawi, Namibia, Timor‐Leste, Ukraine, Zambia, Kenya, Spain, Cameroon, Congo, Serbia, India, Japan, Portugal, South Africa, Viet Nam, Tanzania, Uganda, and Poland) were grouped under Other Nations.

We constructed a flowchart that visually summarizes the major steps of the study to provide a clear overview of the entire research process (Fig 1). For the sequence data, all ASFV genomes were retrieved from the NCBI Genomes database. GenBank files for 235 complete ASFV genomes were downloaded in May 2024 and categorized using the “country” metadata in each file (Fig 2). When country information was not provided, the data were supplemented by reviewing the original publications cited in the GenBank records to assign a provenance. The complete strain dataset is provided in tabular form in Supporting information (S1 Table). Downstream analyses focused on countries for which at least ten genomes were available, enabling robust identification of nation-enriched protein sequence fragments.

### Epitope length determination

We first established the optimal epitope lengths for porcine cytotoxic T lymphocytes (CTL), helper T lymphocytes (HTL), and linear B lymphocytes (LBL) to simplify both the analysis and downstream application of nation-enriched fragments. Epitope data were downloaded from the Immune Epitope Database (IEDB), and the length of each epitope was tabulated to identify candidate lengths suitable for further analysis. In addition, published studies on ASFV multi-epitope vaccine development were reviewed to determine the epitope lengths that are most frequently employed for each lymphocyte subset [9,11,12]. By comparing these two datasets, one or two representative epitope lengths were selected for subsequent sequence analysis: 9 amino acids (aa) for CTLs, 12 aa and 15 aa for HTLs, and 15 aa and 20 aa for LBLs.

### Genome clustering based on unique sequence

Coding‐sequence (CDS) amino-acid sequences were extracted from the downloaded GenBank files using the BioPerl module Bio::SeqIO. Each sequence was then fragmented into 9-, 12-, 15-, and 20-mer peptides using a sliding window with a step size of one residue to ensure that every possible fragment was generated. The redundant fragments were eliminated to obtain a non-redundant (unique) set for each peptide length.

We built a presence/absence matrix to compare genomes and countries; a value of 1 indicated that a unique fragment was present in the CDSs of the genome, whereas 0 indicated its absence (S2 Table in S1 File). Pairwise genomic similarities were calculated using the Euclidean distance, and statistical support for the resulting hierarchical clusters was assessed with the pvclust package in R (version 4.3.1), using 1,000 bootstrap replicates.

### Identification of nation-enriched sequence fragments

For each peptide length (9-mer, 12-mer, 15-mer, and 20-mer), the unique fragments were evaluated for country specificity against the full ASFV genome panel. Statistical significance was assessed in R (version 4.3.1) using Fisher’s exact test. Fragments with an odds ratio > 1 and a *p*-value < 0.05 were designated as nation-enriched fragments and retained for further analyses.

All custom Perl scripts used for sequence processing and statistical analysis in this study have been deposited and are publicly accessible on GitHub (https://github.com/kse0207/Genome-Fragment-Analyzer).

### Epitope prediction

Nation-enriched fragments derived from the five focal countries were pooled and deduplicated before epitope prediction. Cytotoxic T-cell epitopes were predicted using NetMHCpan EL 4.1 against the porcine SLA-1*0401 allele [13], selecting peptides with a percentile rank <0.2. This threshold is considerably more stringent than the commonly recommended ≤1 cutoff by IEDB, aiming to maximize prediction specificity and confidence in the selected candidates. Helper T-cell epitopes were predicted using NetMHCIIpan EL 4.1 with HLA-DRB1*01:01, a surrogate for porcine SLA-DRB1*0201 [14], and retained only peptides with percentile ranks <0.2. Linear B-cell epitopes were predicted using the Kolaskar and Tongaonkar antigenicity method, designating peptides with scores exceeding 1.2 as putative epitopes. This threshold was chosen to ensure higher confidence, as it is more conservative than the typical average-based cutoff of >1.0, as reported in previous studies [15]. The selected candidate epitopes were subsequently carried forward for further *in silico* validation.

### Antigenicity prediction

We used VaxiJen v3.0 for antigenicity prediction to evaluate the antigenicity of the previously selected epitope sequences. The software’s built-in model classifies each peptide as either immunogenic or non-immunogenic and assigns a probability score of 66% or 100%. In this study, sequences predicted as immunogenic with probabilities of 66% or 100% were retained as final epitope candidates.

### Protein origin and functional characterization of selected epitopes

Each epitope sequence was queried against the complete ASFV protein set in the NCBI for Biotechnology Information database using BLASTp to identify its closest homolog and investigate the protein origin and functional characteristics of these candidates. The identified proteins were annotated using UniProt entries and relevant literature to determine their biological functions, roles in viral processes, and immunological significance.

## Results

### Statistics of AA fragments from ASFV genomes

We fragmented each sequence to identify national-enriched regions within the amino acid datasets of the 235 ASFV genomes. The fragmentation scheme was guided by peptide lengths (9-, 12-, 15-, and 20-mers) naturally recognized by immune cells, ensuring that candidate vaccine epitopes were selected on physiologically relevant grounds.

A total of 210,880 fragments (9-mer), 236,880 fragments (12-mer), 259,173 fragments (15-mer), and 265,933 fragments (20-mer) were generated from the complete set of genomes, which displayed a gradual increase in peptide length (Table 1). At the country level, Italy yielded the highest number of nation-enriched fragments: 13,068 (9-mer), 14,654 (12-mer), 16,029 (15-mer), and 17,835 (20-mer). Korea ranked second, with 1,399, 1,439, 1,472, and 1,483 fragments of the respective lengths. China produced 1,068 fragments in the 9-mer set, 1,204 in the 12-mer set, 1,312 in the 15-mer set, and 1,450 in the 20-mer set. Both the Philippines and Russia contributed fewer than 1,000 enriched fragments. In the Philippines, fragments 528 (9-mer), 593 (12-mer), 651 (15-mer), and 721 (20-mer) were observed. Russia showed a distinct pattern; unlike other countries, where fragment counts increased with length, the number of fragments declined as length increased, with 336 (9-mer), 332 (12-mer), 327 (15-mer), and 314 (20-mer) fragments detected.

**Table 1:** Distribution of all the fragments or enriched fragments in each country.

Length (AA)	All Nations (Fragments) ^a^	China (Fragments) ^b^	Italy (Fragments) ^b^	Philippines (Fragments) ^b^	Russia (Fragments) ^b^	South Korea (Fragments) ^b^
9	210,880	1,068	13,068	528	336	1,399
12	236,880	1,204	14,654	593	332	1,439
15	259,173	1,312	16,029	651	327	1,472
20	265,933	1,450	17,835	721	314	1,483

Amino acid sequences were segmented by length, duplicates were removed, and nation-enriched fragments were analyzed and summarized.

a The total number of fragments generated by sequentially slicing each epitope based on its length.

b The number of unique sequences identified in each country from the total fragments.

### Relationship between ASFV genomes

Clustering analysis was performed on all 235 genomes using five (9-, 12-, 15-, and 20-mer) fragment sets to compare genomic similarities and assess how sequences were grouped by country of origin (S1 Fig in S1 File). Italian genomes were separated into two clusters, one of which was highly divergent from the rest of the global genome. The Korean genome formed a single self-contained cluster composed exclusively of 19 genomes detected in Korea, showing no admixture with genomes from other countries. The Philippine genomes were also divided into two groups. One cluster contained genomes from Chinese and several African isolates, whereas the other cluster was distinct. The Russian and Chinese genomes tended to cluster together with genomes from multiple countries. The Chinese genome was divided into four separate groups that were associated with different international clusters. The Russian genome showed close similarity to the genomes of both China and Poland.

### Epitope & antigenicity prediction based on nation-enriched protein fragments

After pooling the national-enriched fragments from the five countries and removing duplicates for each epitope-length class, we obtained 15,876 unique 9-mer peptides for CTL analysis, 19,317 and 21,388 unique fragments for HTL analyses at the 15-mer and 20-mer levels, respectively, and 17,725 and 19,317 unique fragments for 12-mer and 15-mer LBL analyses, respectively. Subsequent epitope prediction identified 136 candidate CTL epitopes from the 9-mer set, 60 and 35 candidate HTL epitopes from the 15-mer and 20-mer sets, respectively, and 57 and 35 candidate LBL epitopes from the 12-mer and 15-mer sets, respectively. The 15-mer LBL set yielded the fewest predicted epitopes (Table 2). Then, an antigenicity prediction was performed to verify the antigenic potential of the selected epitope set. Of the 136 CTL class 9-mer peptides, 39 were predicted to be antigenic. HTL epitopes showed fewer antigenic hits; only 10 of the 60 HTL 15-mers and 8 of the 35 HTL 20-mers surpassed the antigenicity threshold. In contrast, LBL epitopes showed a higher success rate, with 47 of 57 LBL 12-mers and 28 of 33 LBL 15-mers classified as antigenic.

**Table 2:** Result of epitope and antigenicity prediction.

Lymphocyte	Length (AA)	Total fragments ^a^	Epitope seq^b^	Non-Immunogen		Immunogen	
				Probability 66%	Probability 100%	Probability 66%	Probability 100%
CTL	9	18,876	136	59	38	35	4
HTL	15	19,317	60	32	18	6	4
	20	21,388	35	14	13	8	0
LBL	12	17,725	57	8	2	18	29
	15	19,137	33	4	1	10	18

The results from epitope prediction and antigenicity prediction were summarized in a table. For the probability values, the percentages were determined based on the outcomes from the three models used in VaxiJen 3.0: if two models predicted the sequence as immunogen/non-immunogen, it was recorded as 66%, and if all three models predicted the same result, it was recorded as 100%.

a The number of enriched sequences identified in the country (duplicates excluded).

b The number of sequences selected after epitope prediction.

### Identification of epitope distribution and source proteins by country

Ultimately, upon confirming the presence of immunogenic epitopes, we assessed the distribution of nationwide enriched fragments across all 40 selected CTL (9-mer) and 8 HTL (20-mer) epitopes (Table 3). Within the CTL epitope set, 39 nation-enriched fragments were identified, including 33 from Italy, 3 from Korea, 2 from Russia, 1 from China, and 1 from the Philippines. One fragment was shared between Korea and Russia and counted only once. 9 enriched fragments were observed in the 20-mer HTL set: 5 from Italy, one each from Russia and Korea, and one fragment shared among China, Korea, and the Philippines. Among the antigenic 15-mer HTL epitopes, 9 fragments were derived from the 15-mer set, including five from Korea and five from Italy. In the LBL (12-mer) immunogen epitopes, no enriched fragments were found in the Philippines or Russia; instead, 5 fragments were enriched in China, 36 in Italy, and 6 in Korea. The LBL (15-mer) epitopes appeared as country-specific sequences in 4 countries (excluding Russia): 16 were specific to Italy, 8 to Korea, and 1 each to China and the Philippines.

**Table 3:** Country-wise distribution of immunogenic epitopes.

Lymphocyte	Length (AA)	Total seq^a^	Immunogen epitope^b^	China (Fragments) ^c^		Italy (Fragments) ^c^		South Korea (Fragments) ^c^		Philippines (Fragments) ^c^		Russia (Fragments) ^c^	
				66% ^d^	100% ^e^	66% ^d^	100% ^e^	66% ^d^	100% ^e^	66% ^d^	100% ^e^	66% ^d^	100% ^e^
CTL	9	15,876	39	1	0	26	6	3	1	1	0	2	0
HTL	15	19,317	9	0	0	3	1	3	2	0	0	0	0
	20	21,388	8	1	0	5	0	2	0	1	0	1	0
LBL	12	17,725	47	0	5	18	18	0	6	0	0	0	0
	15	19,317	28	0	0	9	10	1	7	1	0	0	0

The table shows the country of origin of the enriched fragments that were selected as immunogen epitopes.

a The number of enriched sequences identified in the country (duplicates excluded).

b The number of sequences selected after epitope & antigenicity prediction.

c Number of fragments selected as immunogen epitopes by country.

d Epitopes predicted to be immunogenic, with an antigenicity probability of 66%.

e Epitopes predicted to be immunogenic, with an antigenicity probability of 100%.

Alignment analysis was used to elucidate the functional origins of the nationwide enriched immunogen fragments. The selected fragments were traced to four principal protein classes: structural proteins, replication/transcription-related proteins, multigene family (MGF) proteins, and hypothetical proteins of unknown function, with those derived from the MGF and hypothetical proteins comprising the majority of the dataset (Table 4).

**Table 4:** Functional and Geographic Characterization of Selected Epitopes. (A)Table summarizing the protein origins and their functional categories for nation-enriched fragments selected as immunogen epitopes. (B) Table showing how fragments in each functional category are distributed across the countries.

(A)					
Protein Function	Protein Name				No. of Fragment
MGF protein	MGF 100-3L, MGF 110-1L, MGF 110-7L, MGF 110-8L, MGF 110-9L, MGF110-11L, MGF 300-4L, MGF 360−1La, MGF 360−2La, MGF 360-10L, MGF 360-13L, MGF 505-4R, MGF_505/530-5R				38
Replication/Transcription	DNA ligase (Chain A), DNA polymerase beta-like protein (Chain A), Helicase/ Putative helicase/primase complex protein, Probable AP endonuclease, Early transcription factor large subunit homolog, Replication, M448R (Putative RNA-ligase)				11
Structural Proteins	Structural protein p17, Structural protein p22, Polyprotein pp220				24
Unknown Function/Hypothetical Proteins	ACD 00270, B407L, C84L, K145R, A528R, CP2475L, I196L, L83L, QP383R, URF48, C129R, DP93R, EP84R, J64R, Lectin-like protein, EGFP,Hypothetical protein				58
(B)					
Protein Function	China (Fragment)	Italy (Fragment)	Philippines (Fragment)	Russia (Fragment)	South Korea (Fragment)
MGF protein	5	10	2	3	21
Replication/Transcription	0	11	0	0	0
Structural Proteins	1	23	0	0	0
Unknown Function/Hypothetical Proteins	1	53	1	1	2

Country-specific analyses revealed a functional distribution. Collectively, replication/transcription- and structural protein–derived fragments were predominantly enriched in Italy, Korean-enriched fragments were almost exclusively MGF-derived, and contributions from China, the Philippines, and Russia were comparatively limited and largely MGF-derived. In the Italian dataset (*n* = 88), 23 fragments (26.1%) originated from structural proteins, 11 (12.5%) from replication/transcription-related proteins, and 54 (61.4%) from hypothetical proteins. The Korean dataset (*n* = 23) yielded 21 fragments (91.3%) from MGF proteins and 2 (8.7%) from proteins of unknown function. China contributed seven fragments, i.e., five (71.4%) from MGF proteins, one (14.3%) from a structural protein, and one (14.3%) from a hypothetical protein. The Philippines (*n* = 3) and Russia (*n* = 4) each supplied fragments exclusively derived from MGF or hypothetical proteins. The complete epitope dataset is provided in tabular form in Supplemental S2 Table in S1 File.

## Discussion

ASFV infections are prevalent in Africa, Europe, and Asia. There are also distinct differences in genomic architecture and sequence characteristics among these regions. This regional genetic heterogeneity arises from the combined influence of multiple factors, including transmission routes, geographic environment, isolation, and environmental selection pressures. For instance, ASFV strains isolated from western Poland shared genetic similarities with those from the eastern region, while displaying unique mutations in specific genes [16]. Similarly, region-specific single nucleotide polymorphisms (SNPs) in ASFV strains detected in wild boars in Korea [17]. These findings suggest that regionally distinct evolutionary trajectories should be critically considered when designing vaccines and diagnostic platforms. Therefore, it is necessary to move beyond single-gene–focused approaches and adopt genome-wide strategies for epitope discovery. In this study, we aimed to address the limitations of previous multi-epitope vaccine development efforts, which have primarily focused on well-characterized structural and functional genes. By performing a genome-wide analysis of CDS across 235 ASFV genomes, we identified a broad set of candidate epitopes and constructed a multi-functional epitope library.

Cluster analysis based on nation-enriched fragments revealed that Italian ASFV genomes formed a distinct and independent cluster, clearly separated from other European and Asian lineages. Most of this cluster consists of samples collected from the island of Italy. This observation is consistent with previous findings, which emphasized the genetic stability and potential monophyletic origin of Sardinian ASFV strains [18,19]. Furthermore, another study suggested that the Sardinian virus underwent gradual local evolution, as evidenced by mutations in specific genomic regions [20]. The viral genomes identified in China and Russia formed mixed clusters with sequences from other countries, suggesting multiple origins and relatively low levels of genetic variation. But ASFV genomes isolated from South Korea are presumed to have been introduced via China, but they exhibited a lower-than-expected level of similarity to Chinese strains. This discrepancy likely reflects selective pressures acting shortly after introduction, possibly owing to biological factors.

Epitope analysis revealed that a substantial proportion of the candidate fragments were derived from multigene family (MGF) proteins. ASFV MGFs are classified into several subfamilies, including MGF 110, 300, 360, and 505/530, which play important roles in viral survival. Members of the MGF360 and MGF505 subfamilies suppress type I interferon production, whereas the MGF110 subfamily plays a crucial role in viral replication and intracellular trafficking [21]. Recently, various studies on vaccine development utilizing these MGF genes have been conducted. Regarding the live-attenuated vaccine strategy via gene deletion, a mutant ASFV derived from the Georgia 2007 strain lacking both MGF360 and MGF505 has been reported to induce robust protective immunity in pigs without causing pathogenicity [22]. Similarly, the co-deletion of CD2v and MGF360-505R has been demonstrated to effectively attenuate viral virulence by suppressing the induction of NF-κB signaling and the transcription of IL-1β in macrophages [23]. Expanding beyond these full-gene deletion approaches, multi-faceted efforts are currently underway to screen precise epitopes within MGFs using *in silico* computational analysis. For instance, recent studies targeting the MGF_110-13L protein identified specific linear epitopes that were found to be highly conserved across diverse variants through comparative sequence analysis of Genotype I and II strains. Furthermore, the integration of *in silico* prediction and experimental validation has successfully revealed that the MGF505-7R protein and specific peptides derived from MGF100-1L serve as core immunodominant antigens commonly recognized by ASFV-specific CD8 + T cells. These findings offer concrete and refined targets for the design of next-generation subunit and multi-epitope-based vaccines capable of eliciting broad cross-protection.

When examined immunogen epitopes by country, notable differences were observed in the distribution of epitope-associated proteins among groups. In countries except Italy, most epitope fragments were derived from MGF-related proteins. In contrast, only a few of fragments from the Italian group originated from MGFs, whereas the remaining were classified as structural, replication-related, or other proteins. This suggests that the geographic isolation of Sardinia may have contributed to the immunogenic enrichment of non-MGF proteins. In addition to these country-specific differences, ASFV genomic fragments exhibiting high variability among countries generally contained fewer predicted immunogenic epitopes than enriched-fragments. This pattern suggests that frequent mutations in these fragments may drive the alteration or loss of antigenic epitopes, resulting in partial immune escape. Similar mechanisms have been reported in rapidly evolving RNA viruses such as SARS-CoV-2, in which recurrent mutations within dominant epitopes lead to reduced predicted immunogenicity and diminished antibody recognition. Although ASFV is a large double-stranded DNA virus with a relatively low mutation rate, previous studies have demonstrated that variations in genes such as CD2v (EP402R), EP153R, and members of the MGF360/505 families can influence immune recognition and protective efficacy. Therefore, the reduced number of immunogenic regions observed in highly variable ASFV fragments may represent an adaptive strategy for evading host immune responses. Taken together, these findings highlight the importance of comprehensively understanding regional differences in ASFV genomic structure and antigenic composition to support the development of broadly effective global vaccines.

Our findings demonstrated that a genome-wide approach can more effectively capture regional genetic diversity and functional complexity, thereby offering broader applicability than gene-restricted strategies. In addition, the fragment library generated in this study encompasses a wide range of functional targets, suggesting its potential utility as a foundational resource for region-specific vaccine design.

In accordance with this research objective, the present phase focused on establishing an analytical strategy that reflects the regional variation and genetic diversity among ASFV strains, and on determining whether antigenic epitopes could be identified through this approach. As a result, vaccine construct design and experimental validation were not performed at this stage but are planned for future follow-up investigations.

## Conclusions

Previous ASFV vaccine research has largely focused on epitope prediction for a limited number of well-characterized viral genes, followed by the design of vaccine candidates based on these epitopes. In contrast, this study comprehensively analyzed the amino acid sequences of all ASFV genomes deposited in NCBI and identified nation-enriched fragments that were uniquely abundant in each country. The epitope and antigenicity prediction of these fragments enabled the proposal of candidate epitopes tailored to specific geographic regions. The resulting dataset provides a foundational resource for vaccine design that reflects nation- and region-specific genetic characteristics. It can be directly leveraged for future ASFV vaccine and diagnostic development. However, because ASFV continues to accumulate novel mutations, it is essential to update the fragment analysis and epitope prediction as new genome sequences become available to keep pace with the rapid evolution of the virus.

## Supporting information

S1 File S1 Table. ASFV genome data list. This table provides detailed information on the NCBI genome datasets analyzed in this study. **S2 Table. Presence/absence matrix of fragment distribution across countries.** Presence (1) and absence (0) of each fragment are indicated for all countries included in the analysis. **S3 Table. ASFV candidate epitope list.** This table stores candidate epitope information identified through epitope prediction and antigenicity analysis**. S1 Fig. Hierarchical clustering of 235 ASFV genomes.** Clustering based on the unique fragments generated at four peptide lengths (9-mer, 12-mer, 15mer, and 20-mer). Hierarchical clusters was assessed with the pvclust package in R (bootstrap = 1,000).
(ZIP)
