# Supplementary material for: Identification of Candidate epitopes from nation-enriched sequences in the African swine fever virus genomes
Source: PLoS One. 2026 Jul 23;21(7):e0354143. doi: 10.1371/journal.pone.0354143 (PMC13395360; doi:10.1371/journal.pone.0354143)
Supplement: S1 File — This table provides detailed information on the NCBI genome datasets analyzed in this study. S2 Table. Presence/absence matrix of fragment distribution across countries. Presence (1) and absence (0) of each fragment are indicated for all countries included in the analysis. S3 Table. ASFV candidate epitope list. This table stores candidate epitope information identified through epitope prediction and antigenicity analysis. S1 Fig. Hierarchical clustering of 235 ASFV genomes. Clustering based on the unique fragments generated at four peptide lengths (9-mer, 12-mer, 15mer, and 20-mer). Hierarchical clusters was assessed with the pvclust package in R (bootstrap = 1,000). (ZIP) [file pone.0354143.s001.zip › S1_fig.pdf]

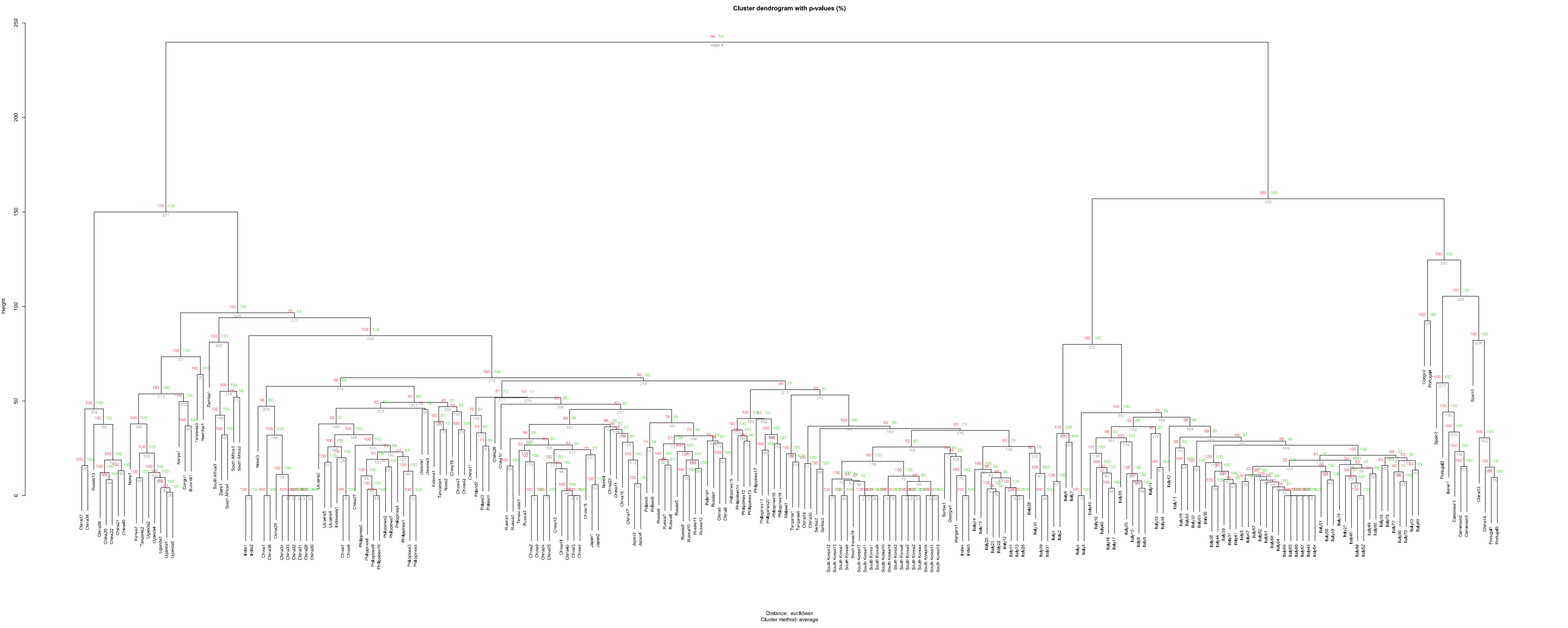

Cluster dendrogram with p-values (%)
au
bp
edge #
Height
250
200
150
100
50
0
Distance: euclidean
Cluster method: average

Fig. S1 Hierarchical clustering of 235 ASFV genomes. Clustering based on the unique fragments generated at four peptide lengths (9-mer, 12-mer, 15mer, and 20-mer). Hierarchical clusters was assessed with the pvclust package in R (bootstrap = 1,000)
